# A novel loop-mediated isothermal amplification-lateral flow dipstick method for *Helicobacter pylori* detection

**DOI:** 10.3389/fmicb.2023.1094600

**Published:** 2023-03-23

**Authors:** Wenwen Liu, Gang Lu, Yu Wang, Zhenghong Chen, Yunyun Gao, Zhipeng Yin, Yi Wu, Xiaoqian Lv, Pengbo Guo, Yinghui Zhao

**Affiliations:** ^1^Department of Clinical Laboratory Medicine, The First Affiliated Hospital of Shandong First Medical University and Shandong Provincial Qianfoshan Hospital, Shandong Medicine and Health Key Laboratory of Laboratory Medicine, Jinan, Shandong, China; ^2^Department of Pathogen Biology, School of Clinical and Basic Medical Sciences, Shandong First Medical University and Shandong Academy of Medical Sciences, Jinan, Shandong, China; ^3^Department of Clinical Microbiology Laboratory, Shandong Provincial Hospital Affiliated to Shandong First Medical University, Jinan, Shandong, China; ^4^Key Laboratory of Medical Microbiology and Parasitology, Department of Microbiology, Guizhou Medical University, Guiyang, China

**Keywords:** *Helicobacter pylori*, ureB gene, loop-mediated isothermal amplification, lateral flow dipstick, novel detection method

## Abstract

**Introduction:**

To eradicate *Helicobacter pylori* (*H. pylori*) and reduce the risk of gastric cancer, a sensitive, specific, convenient, and simple detection method is needed. This study aimed to establish a novel loop-mediated isothermal amplification-lateral flow dipstick (LAMP-LFD) method for *H. pylori* detection.

**Methods:**

LAMP primer design software was used to design primers for the conserved sites of the *H. pylori* ureB gene. UreB-FIP-labeled biotin was used for LAMP amplification, and FAM-labeled probes were specifically hybridized with LAMP amplification products, which were then detected by LFD. In addition, a clinical study was conducted to assess LAMP-LFD in 20 fecal samples.

**Results:**

The results of the optimization indicated that *H. pylori* could be specifically detected by LFD without cross-reaction with other non-*H. pylori* bacteria when the LAMP was performed at 65°C for 60 min. The lower limit of the detection method was 10^2^ copies/μL, which was 100 times the sensitivity of polymerase chain reaction (PCR). *H. pylori*-positive fecal samples were detected by LAMP-LFD in 13/20 patients.

**Discussion:**

In conclusion, a new LAMP-LFD assay has been fully established and confirmed for *H. pylori* detection. The entire process can be completed in approximately 1.5 h, with the advantages of strong specificity, high sensitivity, and simple operation. This study provides a novel potential method for the detection of *H. pylori* in the clinical settings of primary hospitals and low-resource countries.

## Introduction

*Helicobacter pylori* (*H. pylori*), defined as a class I carcinogen by the World Health Organization, is the most common pathogen that infects the stomach and often resides on the surface of the pylorus and duodenal mucosa (Hooi et al., 2017). More than 50% of the world's population is infected with it, and the infection rate in developing countries is generally higher than that in developed countries (Roszczenko-Jasińska et al., 2020). It can cause various gastrointestinal and extragastric diseases, such as gastritis, gastric ulcers, gastric cancer, mucosa-associated lymphoid tissue lymphoma, Alzheimer's disease, and other neurological or blood diseases (Kountouras et al., 2010; Kutlubay et al., 2014; Sacc et al., 2014; Sarem and Corti, 2016; Muhammad et al., 2017; Zamani et al., 2017; Mladenova, 2019; Pellicano et al., 2020; Howden, 2021). Early eradication of the bacterium is important to prevent and control these diseases. Therefore, it is crucial to develop a rapid, accurate, and suitable method for *H. pylori* detection.

Techniques for *H. pylori* detection can be classified into invasive and non-invasive approaches (Mohammadian and Ganji, 2019). Rapid urease test (RUT), histological examination, and bacterial isolation or culture are considered invasive methods, while PCR is considered a non-invasive method (Di Bonaventura et al., 2004; Patel et al., 2014; Milani et al., 2019; Abdelmalek et al., 2022). Each approach has its advantages and limitations. Of the non-invasive methods, PCR has good sensitivity and specificity; however, it requires PCR and gel imaging equipment, limiting its use in primary hospitals or low-resource countries. To overcome these difficulties, a new method must be developed.

Loop-mediated isothermal amplification (LAMP) is a novel gene amplification strategy for continuous extension and replacement of four primer pairs within 1 h under certain temperature conditions and strand displacement by Bst DNA polymerase (Liu et al., 2019). This method has many advantages, including simplicity, speed, high sensitivity and specificity, and minimal equipment or reagent requirements (Domesle et al., 2018). The amplification products of LAMP are mainly detected by turbidimetry, electrophoresis, or calcein fluorescent dyes (Bakhtiari et al., 2016, 2019; Park et al., 2018; Sohrabi et al., 2021). The contact of ethidium bromide (EB), a carcinogen, the potential harm from calcein fluorescent dye to the human body, and the subjectivity of the turbidimeter reduce the practicality of LAMP in the field (Zhang et al., 2019). Lateral flow dipstick (LFD) can overcome these limitations (Lalle et al., 2018). Consequently, this study combined LAMP with LFD to establish a LAMP-LFD method for the detection of *H. pylori*, which will provide a reliable and effective diagnostic tool for *H. pylori* infection.

## Materials and methods

### Experimental materials

A bacterial DNA extraction kit was obtained from Dalian Bao Biotechnology Co., Ltd. A PCR amplification kit and a Bst DNA polymerase were obtained from Kangwei Century Biotechnology Co., Ltd. Hydroxynaphthalene blue (HNB) was obtained from Sigma Co., Ltd. The dNTP mixture was obtained from SOLEBAO Biotechnology Co., Ltd. A ^14^C Urea breath test (UBT) kit was obtained from Shenzhen Zhonghe Haidewei Biotechnology Co., Ltd. A 5kb DNA marker was obtained from Nuovizan Biotechnology Co., Ltd. *H. pylori* (ATCC26695), *Escherichia coli* (DH5α), and *Pseudomonas aeruginosa* (BNCC186335) were preserved in our laboratory. *Staphylococcus aureus* was presented by Jing Ni, a teacher from the School of Public Health of Shandong First Medical University. The fecal samples of patients with gastric diseases were collected from the Oncology Department of Taishan Hospital. The fecal samples experiments were approved by the Ethics Committee of Shandong First Medical University (R202211250164).

### Bacterial culture and DNA extraction

*H. pylori* (ATCC26695) was cultured on a yolk agar medium under the conditions of 85% N_2_, 10% CO_2_, and 5% O_2_ at 37°C for 3 d. Other bacteria (*Escherichia coli, Pseudomonas aeruginosa*, and *Staphylococcus aureus*) were cultured on ordinary agar medium at 37°C for 20 h under the same gas conditions. Bacterial DNA was extracted according to the kit (TaKaRa MiniBEST Bacteria Genomic DNA Extraction Kit Ver.3.0, TaKaRa) instructions. The concentration and purity of the DNA were evaluated using a NanoDrop UV spectrophotometer with A_260_ and A_280_ absorbance values.

### Primer and probes

The *H. pylori* ureB gene sequence was obtained from NCBI (sequence number: WP_ 000724295.1, AFV41291.1, and AY714224.1). The conserved region of the ureB gene was selected by Mega and Bio edit software. Primer Explorer V5 was used to design several groups of LAMP primers and probes, and a group with high specificity was selected. Each group of primers and probes included two internal primers (FIP, 5′-CCAGCACCTTCAGTGTGGAAATTGCGTGGAAGACACTATGG-3′ and BIP 5′ -GGCGGACACGCTCCTGATATTTGGAAGCGGGAAGAATGTTG-3′), two external primers (F3, 5′-GTCGCTATCCACACAGACAC-3′ and B3, 5′-AGTGAAAGGGATAGTGGGGT-3′), and a probe (Hp, 5′-CCGGACGCACTATGCACAC-3′) in which the 5′ end of FIP was labeled with biotin, while the 5′ end of Hp was labeled with FAM. The primers and probes were synthesized and labeled by Bioengineering Co., Ltd. (Shanghai, China). PCR primers (UreB-F, 5′-GAACATGACTACACCAT-3′ and UreB-R, 5′-TGGTTTGAGGGCGAATC-3′) were synthesized by the aforementioned company.

### Establishment and optimization of the LAMP reaction system

The 25 μL reaction system consisted of 2.5 μL 10× reaction buffer, 6 mM MgSO_4_, 1.4 mM dNTPs, 1.6 μM FIP, 1.6 μM BIP, 0.2 μM F3, 0.2 μM B3, 0.32 U/μL Bst DNA polymerase, 1 μL *H. pylori* DNA template, and 11.5 μL ddH_2_O. The solutions were added to a 200 μL reaction tube. The tubes were placed in a 65°C water bath for 1 h, followed by a 85°C water bath for 20 min. Reaction products were verified by 2.5% agarose gel electrophoresis.

Next, the LAMP reaction conditions were optimized. The dNTP mixture concentration was tested from 4 mM to 14 mM, with the concentration increased at 2 mM intervals. The LAMP amplification products were analyzed using gel electrophoresis. The dNTP concentration with the most obvious electrophoretic band was selected to optimize the Mg^2+^ content in the reaction. The Mg^2+^ content was tested from 0 to 3.0 μL at 0.5 μL intervals. LAMP amplification was performed, and the products were detected using gel electrophoresis. Bst DNA polymerase content was optimized according to the dNTP and Mg^2+^ optimized reaction system. Bst DNA polymerase content was tested from 0.5 to 2.0 μL at 0.5 μL intervals. LAMP amplification was performed, and the products were observed after gel electrophoresis. The concentration ratio of the internal and external primers was then optimized. The concentration of external primers (F3, B3) was maintained at 5 μM, while the concentrations of internal primers (FIP and BIP) were diluted to 50, 40, 20, 10, and 5 μM; the concentration ratios of internal and external primers were 10:1, 8:1, 4:1, 2:1, and 1:1, respectively. After the reaction, agarose gel electrophoresis was performed for verification purposes. Based on the earlier optimized reaction system, the reaction time and temperature were optimized. The reaction temperatures were tested at 50, 55, 60, 65, and 70°C. The reaction time was set from 15 to 75 min at 15-min intervals. The optimal reaction temperature and time were selected to correspond to the most obvious electrophoresis band using 2.5% gel electrophoresis analysis.

### Establishment of LAMP-LFD

The optimized LAMP reaction system was then used with the biotin-labeled FIP and FAM-labeled probe. The reaction system included a 1 μL FAM-labeled probe (20 pmol/L), hybridized at 65°C for 5 min, and then heated at 85°C for 20 min. The reaction products (9 μL) were added to 190 μL ddH_2_O, and the diluted reaction product was added to the sample area of the LFD. The product migrated forward to the sample-binding pad, detection line, and quality control line. The DNA probe labeled by biotin-FAM effectively bound to streptavidinized colloidal gold on the sample-binding pad. If biotin-FAM probe amplification of the target gene occurred, the product would be captured by the anti-FAM antibody to form a ternary complex, which would show a red band on the detection line. In contrast, biotin-FAM-labeled probes that do not form hybrid products would cross the detection line and combine with biotinylated BSA on the quality control line, showing only red bands on the quality control line.

### Analysis of specificity, sensitivity, and repeatability of LAMP-LFD

To verify the specificity of LAMP-LFD, DNA from *H. pylori, Escherichia coli, Pseudomonas aeruginosa*, and *Staphylococcus aureus* was extracted. The optimal LAMP assay was used for amplification. Amplification products were detected using LFD and 2.5% agarose gel electrophoresis.

To test the sensitivity of the method, the concentration of *H. pylori* DNA was diluted with sterile ddH_2_O with a 10-fold gradient for a final concentration range of 10–10^8^ copies/μL. Different concentrations of DNA were used as templates to perform the optimized LAMP amplification reaction, and the amplification products were detected using LFD and 2.5% agarose gel electrophoresis. At the same time, PCR was conducted using the following reaction system: 25 μL 2× Es Taq Master Mix (Dye), 2 μL ureB-F primer, 2 μL ureB-R primer, 1 μL template DNA, and 20 μL ddH_2_O. Cycling conditions were as follows: denaturation at 94°C for 2 min, 30 cycles of denaturation at 94°C for 30 s, annealing at 56°C for 30 s, elongation at 72°C for 30 s, and final elongation at 72°C for 2 min. The PCR products were detected using 1.5% agarose gel electrophoresis.

Three groups of parallel samples were prepared to test the repeatability of this method. *H. pylori* DNA was diluted to 10^4^ copies/μL using ddH_2_O. The optimized LAMP reaction system was prepared using diluted templates. Finally, LFD and 2.5% agarose gel electrophoresis were used to detect the amplification products in the three groups of samples.

### The detection of clinically isolated strains by LAMP-LFD

Fecal samples from 20 patients with gastric disease were collected. *H. pylori* DNA from feces was extracted using the kit. LAMP-LFD was used to detect bacterial DNA. PCR and ^14^C-urea breath test (^14^C-UBT) were conducted simultaneously.

## Results

### Optimization of the LAMP reaction system

**Figure 1:**
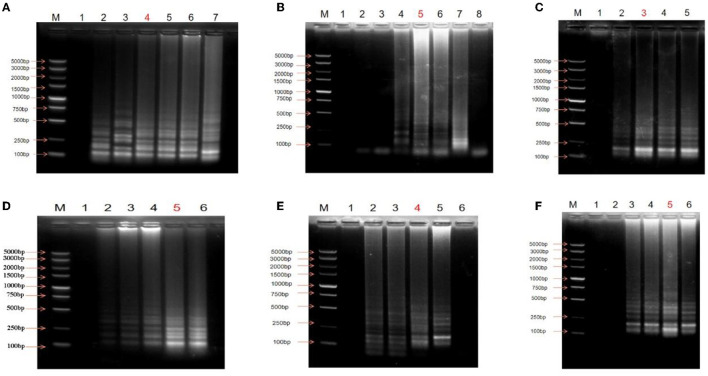
Optimization of the LAMP reaction. **(A)** Optimization of dNTPs concentration, M: DNA Maker DL5000; lane 1: Negative control; lane 2: 4 mM; lane 3: 6 mM; lane 4: 8 mM; lane 5: 10 mM; lane 6: 12 mM; lane 7: 14 mM. **(B)** Optimization of Mg^+^ content, M: DNA Maker DL5000; lane 1: Negative control; lane 2: 0 μL; lane 3: 0.5 μL; lane4: 1.0 μL; lane 5: 1.5 μL; lane 6: 2.0 μL; lane 7: 2.5 μL; lane 8: 3.0 μL. **(C)** Optimization of enzyme content, M: DNA Marker DL5000; lane 1: Negative control; lane 2: 0.5 μL; lane 3: 1.0 μL; lane 4: 1.5 μL; lane 5: 2.0 μL. **(D)** Optimization of primer concentration ratio (internal primer: external primer), M: DNA Marker DL5000; lane 1: Negative control; lane 2: 1:1; lane 3: 2:1; lane 4: 4:1; lane 5: 8:1; lane 6: 10:1. **(E)** Optimization of reaction temperature, M: DNA Maker DL5000; lane 1: Negative control; lane 2: 50°C; lane 3: 55°C; lane 4: 60°C; lane 5: 65°C; lane 6: 70°C. **(F)** Optimization of reaction time, M: DNA Maker DL5000; lane 1: Negative control; lane 2: 15 min; lane 3: 30 min; lane 4: 45 min; lane 5: 60 min; lane 6: 75 min. The lanes marked red are the optimal conditions.

The concentration of dNTPs was optimized first. The target sequence was amplified when the dNTP concentration was 4–14 mM (Figure 1A); bands were unclear when the concentration was lower than 4 mM. When the dNTP concentration was 8 mM, the electrophoretic band exhibited an obvious ladder shape. The electrophoretic band did not change significantly when the concentration was between 8 and 10 mM. Consequently, 8 mM was selected as the optimal concentration of dNTPs. Optimization of the Mg^2+^ concentration was then performed. There were no bands in the 0 and 0.5 μL Mg^2+^ groups (Figure 1B). When the Mg^2+^ concentration was 1–2.5 μL, the target bands were observed, and the band of the 1.5 μL group was the most intense. The target band was not detected when the Mg^2+^ concentration was 3.0 μL; the specificity of the reaction might have been reduced due to the high Mg^2+^ content. Therefore, it was determined that the most suitable Mg^2+^ concentration in the reaction system was 1.5 μL.

Enzyme concentration optimization experiments showed that the electrophoresis band was bright and clear when the enzyme concentration was 1.0 μL (Figure 1C). However, with increasing enzyme concentration, the brightness and clarity of the electrophoresis bands did not change. Consequently, 1 μL of Bst DNA polymerase was found to be the optimal concentration in the reaction system.

The LAMP reaction was performed to optimize primer concentration, and the electrophoresis results revealed that bands were not obvious when the concentration ratio of the internal and external primers was 1:1 (Figure 1D). The brightness and clarity of the bands gradually improved with an increase in the ratio of the internal and external primers; the bands showed the highest intensity when the ratio was 8:1. This did not increase further when the concentration ratio of the internal and external primers was increased to 10:1. Therefore, the internal primer and external primer concentrations were determined to be 40 μM and 5 μM, respectively (the ratio of the internal and external primers was 8:1).

The results of electrophoresis following temperature optimization showed that target bands were observed when the reaction temperature was 50– 65°C, whereas no target bands were found after electrophoresis when the reaction temperature rose to 70°C (Figure 1E). There was no significant difference between 50 and 55°C, and the bands were dim because the amplification efficiency of the reaction was low. When the temperature was increased to 60°C, the bands were bright. However, the 65°C group had the brightest bands, indicating that 65°C was the optimal reaction temperature.

Finally, reaction time was optimized. No target bands were observed when the reaction time was 15 min (Figure 1F). The bands were the brightest and presented an obvious ladder shape when the reaction time was increased to 60 min. Therefore, 60 min was selected as the optimal time.

### Establishment of LAMP-LFD

**Figure 2:**
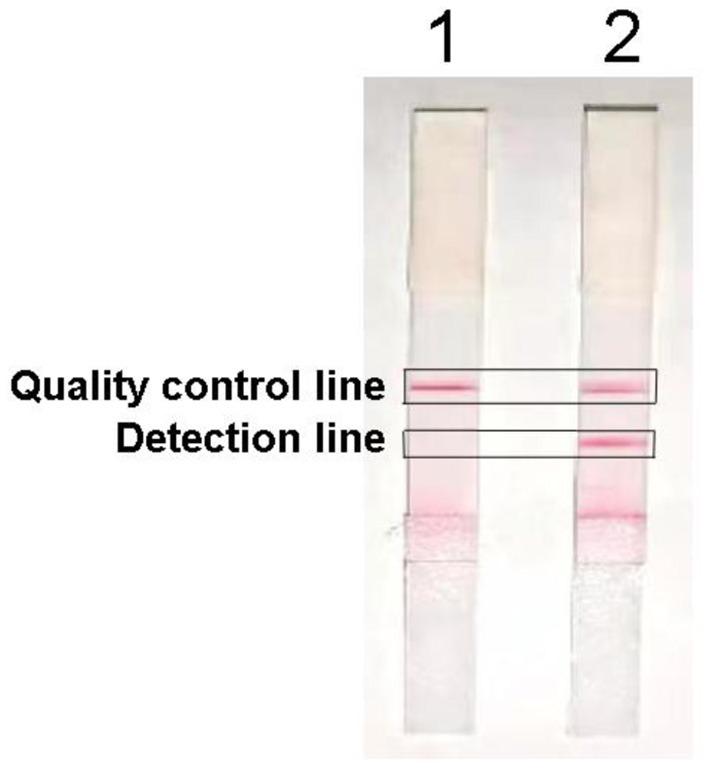
Establishment of LAMP-LFD for *H. pylori*, lane 1: Negative control; lane 2: sample detection.

The positive sample showed red bands on both the detection line and the quality control line, whereas the negative sample only showed red bands on the quality control line (Figure 2), indicating that the LAMP-LFD was successfully established.

### Specificity of LAMP-LFD

**Figure 3:**
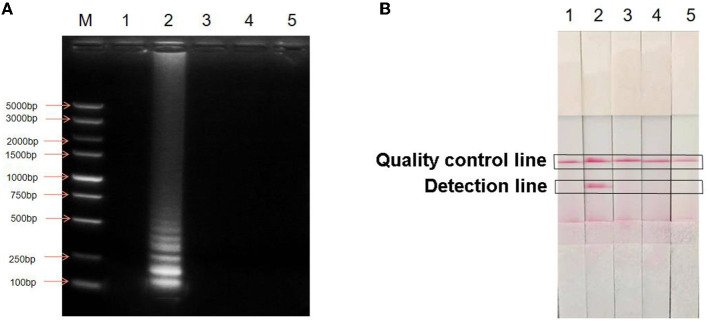
Specific analysis of LAMP-LFD for *H. pylori*. **(A)** Detection of agarose gel electrophoresis; **(B)** Detection by LAMP-LFD; M: DNA Maker DL5000; lane 1: Negative control; lane 2: *H. pylori*; lane 3: *Escherichia coli*; lane 4: *Staphylococcus aureus*; lane 5: *Pseudomonas aeruginosa*.

The established LAMP-LFD assay was used to detect *E. coli, S. aureus*, and *P. aeruginosa* (Figure 3). When *H. pylori* DNA was used as the template, the detection and quality control lines of the horizontal chromatography strip showed two obvious red bands and the electrophoresis result showed typical ladder-shaped bands, indicating a positive detection result. In contrast, no electrophoretic bands were observed when ddH_2_O and other bacterial DNA samples were used as templates. Simultaneously, no red bands were observed on the detection line of the horizontal chromatographic test strip, indicating that the test result was negative. The results showed that the established LAMP-LFD had good specificity.

### Sensitivity of LAMP-LFD

**Figure 4:**
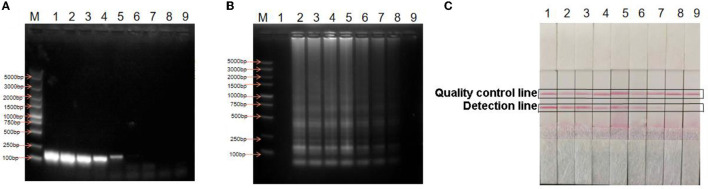
Detection of sensitivity. **(A)** Sensitivity of PCR; **(B)** Sensitivity of LAMP agarose gel electrophoresis; **(C)** Sensitivity of LAMP-LFD; M: DNA Marker DL5000; lane 1: Negative control; lane 2: 10^8^copies/μL; lane 3: 10^7^ copies/μL; lane 4: 10^6^ copies/μL; lane 5: 10^5^ copies/μL; lane 6: 10^4^ copies/μL; lane 7: 10^3^ copies/μL; lane 8: 10^2^ copies/μL; lane 9: 10^1^ copies/μL.

*H. pylori* DNA was diluted with a 10-fold gradient to obtain a DNA concentration of 10 to 10^8^ copies/μL. LAMP was performed under the optimized conditions, and the reaction products were detected using LFD and agarose gel electrophoresis (Figures 4B, C). The lowest detection concentration was 10^2^ copies/μL. The same concentration of DNA was used to perform the PCR (Figure 4A). The lowest concentration detected using PCR was 10^4^ copies/μL. The concentration of DNA detected by LAMP-LFD was 100 times higher than that detected by PCR. Therefore, the LAMP-LFD method established in this study could be used for *H. pylori* detection.

### Repeatability of LAMP-LFD

**Figure 5:**
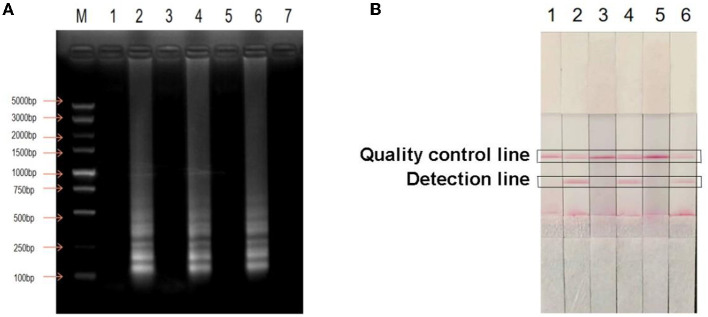
Test of repeatability. **(A)** Test of LAMP agarose gel electrophoresis repeatability; **(B)** Repeatability detection by LAMP-LFD; M: DNA Marker DL5000; lane 1: Negative control; lane 2: *H. pylori* DNA; lane 3: Negative control; lane 4: *H. pylori* DNA; lane 5: Negative control; lane 6: *H. pylori* DNA.

In total, three samples of *H. pylori* DNA were separately diluted to 10^4^ copies/μL and used to perform LAMP assays. The amplified products were detected using LFD and electrophoresis. Positive results were obtained for the three parallel reactions (Figure 5), indicating that LAMP-LFD was repeatable.

### Clinical sample detection by LAMP-LFD

The established LAMP-LFD, PCR, and ^14^C-UBT assays were used to simultaneously detect *H. pylori* in 20 clinical fecal samples. A total of 13 *H. pylori*-positive cases were detected by LAMP-LFD, 11 by PCR, and 15 by ^14^C-UBT.

## Discussion

*H. pylori* is known to be the most important biological factor of gastric cancer, with high infection and recurrence rates worldwide (Yan and Jun, 2019). Early detection of *H. pylori* infection, fast treatment, and prevention of its transmission to other people are important strategies to reduce its harm. Currently, numerous detection methods have been established, but each diagnostic method has one or more disadvantages, such as difficult operation, time consumption, insufficient sensitivity or specificity, expensive instruments or reagents, harm to the human body, and environmental pollution (Ju and Kim, 2015; Wang et al., 2015; Syahniar et al., 2019). Therefore, it is necessary to develop new methods for overcoming these limitations.

The LAMP-LFD method described here is the first of its kind for the detection of *H. pylori* infection. The ureB gene of the bacterium was used as the target gene to establish this method. Specific LAMP primers and LFD detection probes were designed, and the reaction parameters were optimized. The LAMP assay relies on four specific primers for the identification of six gene regions in the target gene without non-specific amplification, compared with PCR using two primers to complete nucleic acid amplification (Liu et al., 2019). Previous studies have shown that the primer, Mg^2+^, and enzyme concentration can directly affect the specificity and sensitivity of the detection method (Mori et al., 2001). Therefore, the reaction parameters of LAMP were optimized to ensure the high specificity and sensitivity of the method.

The LAMP-LFD assay detected a minimum concentration of *H. pylori* of 10^2^ copies/μL, which suggested that its sensitivity was 100 times that of PCR. LAMP-LFD amplification products were detected using a horizontal chromatography strip with probe markers. Negative or positive results were judged according to the presence of a red strip at the detection line. The detection method could be performed without calcein and hydroxynaphthalene blue dyes, eliminating errors caused by subjective color changes and reducing contact with carcinogenic EB when preparing agarose gel electrophoresis (Domesle et al., 2018; Bakhtiari et al., 2019). In summary, the method takes advantage of the high sensitivity and specificity of LAMP, as well as the ease of display and safety of LFD. Although various molecular detection methods for *H. pylori* have been developed in recent years (Molnar et al., 2008; Taborda et al., 2018; Ji et al., 2020), these methods require special instruments, such as PCR, fluorescent quantitative PCR, and high-throughput sequencing, which restrict the implementation of *H. pylori* detection in primary hospitals or low-resource countries. Compared with other molecular methods, LAMP-LFD has the advantages of simplicity, fast operation, minimal experimental equipment requirements, less consumables, and intuitive results.

Fecal samples were analyzed by LAMP-LFD, PCR, and ^14^C-UBT. The positive detection rate of LAMP-LFD was higher than that of PCR and lower than that of ^14^C-UBT. The LAMP-LFD method established is a fast, simple, effective, specific, and sensitive technique for the identification of *H. pylori*. Therefore, LAMP-LFD may substitute PCR and ^14^C-UBT for the detection of *H. pylori* infection. Furthermore, this method avoids environmental contamination and harm caused by radioactive ^14^C in children. In addition, it is a non-invasive approach and is applicable to almost all groups, such as the elderly, children, and people with respiratory diseases.

## Conclusion

In conclusion, a LAMP-LFD method for *H. pylori* detection was successfully developed in this study. This method has outstanding accuracy, high specificity, and sensitivity and is simple, fast, and independent of expensive instruments or reagents. The entire process can be completed within 1.5 h, depending only on the water bath and the test strip. It has been developed for the selective detection of *H. pylori* in feces, which is a non-invasive detection method suitable for use in primary hospitals or low-resource countries. This study provides novel strategies for *H. pylori* detection that are worth popularizing.

## Data availability statement

The datasets presented in this study can be found in online repositories. The names of the repository/repositories and accession number(s) can be found in the article/supplementary material.

## Author contributions

YZ and PG conceived and designed the study and critically revised the manuscript. WL carried out the experiments. GL drafted the manuscript. YWa, ZC, YG, ZY, YWu, and XL contributed to the revision of the manuscript. All authors read and approved the final manuscript.
